# Comprehensive identification and potential application of genetic alteration-driven enhancer RNAs for eRNA-targeted therapy in breast cancer

**DOI:** 10.1016/j.gendis.2023.101124

**Published:** 2023-09-23

**Authors:** Hongying Zhao, Caiyu Zhang, Lin Bo, Lixia Wang, Wangyang Liu, Yaopeng Shu, Kailai Liu, Ying Liu, Meiting Fei, Li Wang

**Affiliations:** a College of Bioinformatics Science and Technology, Harbin Medical University, Harbin, Heilongjiang 150081, China; b Ninth People's Hospital, Shanghai Jiao Tong University School of Medicine, Shanghai 200125, China; c Shanghai Institute of Precision Medicine, Shanghai 200125, China

Enhancer RNAs (eRNAs) are a class of non-coding RNA, which play a critical role in tumor progression.^1^ Previous studies have indicated abundant CpG methylation, somatic mutation, and copy number variation in eRNA regions in cancers.^2^^,^^3^ We constructed a landscape of genetic alteration-driven eRNAs and provided an integrative pipeline to identify drug candidates that affect eRNA activity. Furthermore, we explored the prognostic value of genetic alteration-driven eRNAs.

In this study, we generated an integrative pipeline to identify genetic alteration-driven eRNAs in BRCA (breast cancer) by integrating somatic mutation, DNA methylation, copy number variation, and TCeA (The Cancer eRNA Atlas, https://bioinformatics.mdanderson.org/public-software/tcea) eRNA expression data (Fig. 1; Fig. S1). We constructed circular maps to obtain a global overview of 431 genetic alteration-driven eRNAs in BRCA (Fig. 1A). Notably, six eRNAs significantly overlapped with known BRCA enhancers (*P* = 0.02; hypergeometric test; Fig. 1B). For example, eRNA (chr20:46700957–46701130) is a known risk factor for BRCA, and its known target SULF2 showed significant up-regulation and positive correlation with two eRNAs (chr10:9115735–9116358 and chr10:8903701–8904023) that are transcribed from the same super-enhancer region positively correlated with their target gene GATA3. Three eRNAs showed copy number amplification and up-regulated expression in BRCA (Fig. 1C). eRNAs could induce oncogenes expression and may be considered potential anticancer drug targets.^2^^,^^4^ We generated an integrative pipeline to identify candidate small molecules that can affect genetic alteration-driven eRNA activity. In total, there were 1139 predicted drug–eRNA pairs involving 234 eRNAs and 343 drugs (Fig. 1D). Specifically, there were 443 predicted activating drug–eRNA pairs and 696 inhibiting drug–eRNA pairs. Most of the shortest path length values of the predicted drug–eRNA pairs were 2, which means that our predictions were not random and that the predicted drugs might disrupt eRNAs by targeting regulatory or interacting cofactors (Fig. S2, 3).

**Figure 1:**
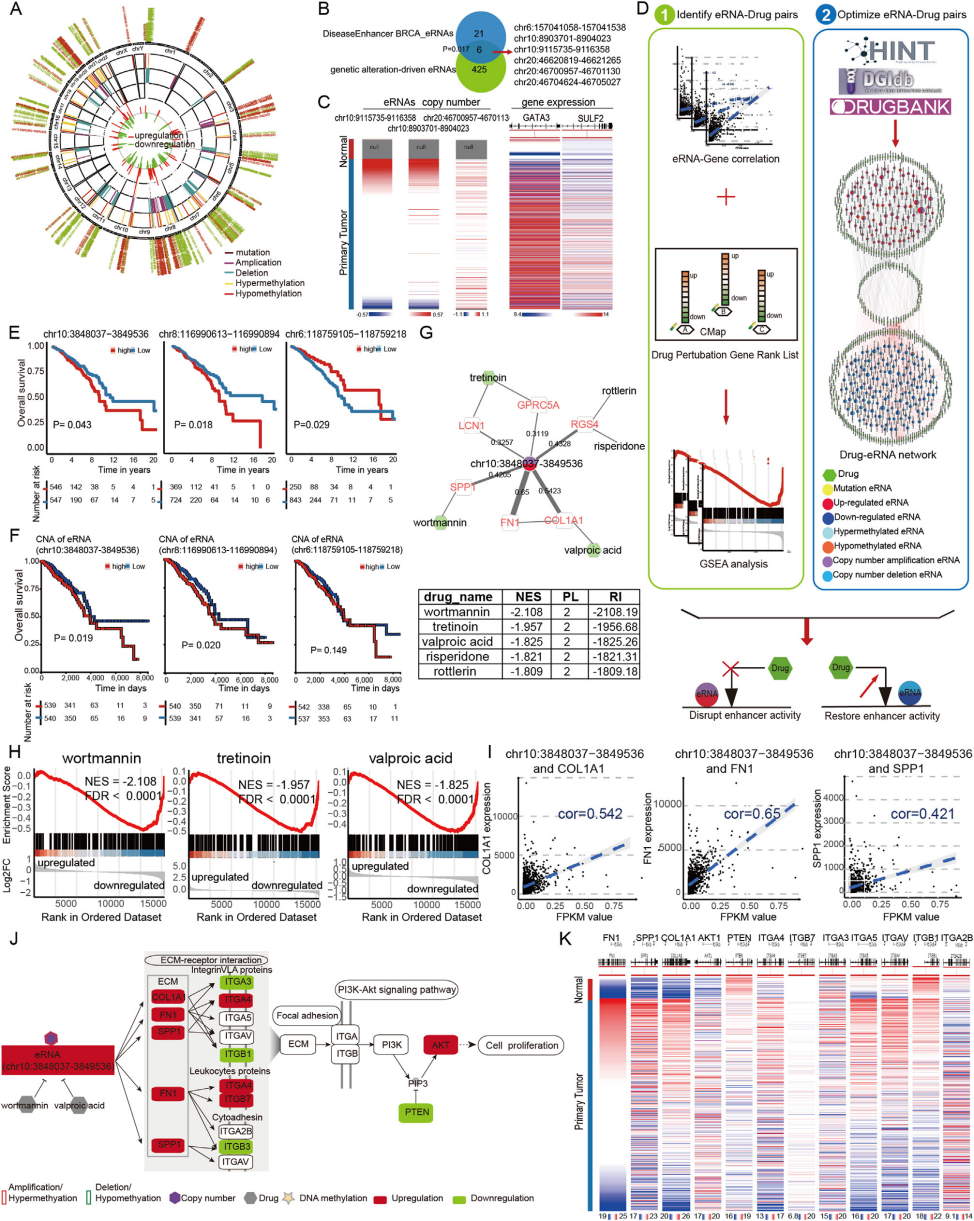
Genetic alteration-driven eRNAs in breast cancer. **(A)** A landscape of eRNAs with differential genetic alterations in breast cancer. The circular map consists of four circles. From the outermost circle map to the innermost circle map represents differential methylation, copy number variation, and mutation sites in the eRNA region. The positions of peaks in the innermost circle correspond to the differential expression of eRNAs, and the height of the peaks at eRNA is proportional to the differential expression level. **(B)** Overlapping of genetic alteration-driven eRNAs in breast cancer with known breast cancer enhancers from DiseaseEnhancer. **(C)** Copy number map of eRNAs and expression profile of their target genes. **(D)** Identification of small molecules that affect genetic alteration-driven eRNAs. Step 1: Gene set enrichment analysis was used to identify genetic alteration-driven eRNAs that are significantly affected by drug interference. Step 2: We optimized eRNA–drug pairs by integrating the standardized enrichment score of GSEA analysis and the standardized shortest path length between drugs and eRNAs in the biological network. **(E)** The Kaplan–Meier survival curve according to eRNA expression. **(F)** The Kaplan–Meier survival curves based on the copy number of eRNAs. **(G)** Network visualization illustrating path lengths from the eRNA to five candidate drugs for eRNA inhibition. The colors of the text represent differential expression, with red for up-regulation and green for down-regulation. The thickness of the edge represents the correlation coefficient between the two vertices. The fill color of the node represents the genetic alteration of eRNAs. The corresponding table shows metrics that describe each of these drugs in relation to eRNA. The normalized enrichment scores were obtained from GSEA; the PL is the shortest network path length required to connect eRNA to the drug; and the RI is the prediction score determined by integrating the normalized enrichment scores and the standardized shortest path lengths. **(H)** The results for the GSEA to calculate whether the target genes of eRNAs are significantly affected by drug perturbations based on CMap. **(I)** Expression correlation between eRNAs and their target genes. **(J)** The disturbance of biological pathways affected by genetic alteration-driven eRNAs and their related genes contributing to breast cancer survival. The polygon indicates the genetic alteration including copy number and DNA methylation. The node color corresponds to the expression difference. **(K)** The expression profiles of eRNA-related genes in the biological pathways in breast cancers.

To characterize the prognostic value of genetic alteration-driven eRNAs in BRCA, we analyzed the expression of 431 genetic alteration-driven eRNAs in 1094 patients (Table S1). Univariate Cox proportional hazards analysis showed that age, HER2 status, TNM stage, AJCC pathological stage, and six genetic alteration-driven eRNAs were significantly associated with overall survival (Table S2). We performed a multivariate Cox proportional hazards model analysis on the expression of the six eRNAs in relation to clinical parameters. We identified three eRNAs as independent risk factors for BRCA prognosis (Fig. S4A and Table S3). The Kaplan–Meier survival curve showed that higher expression of two eRNAs (chr10:3848037–3849536 and chr8:116990613-116990894) and lower expression of one eRNA (chr6:118759105-118759218) were associated with poorer prognosis (log-rank test, Fig. 1E). Furthermore, we characterized the prognostic value of copy number variations of eRNAs. We identified two eRNAs (chr10:3848037–3849536 and chr8:116990613-116990894) whose copy number amplifications were significantly associated with a poor prognosis (log-rank test; Fig. 1F). For example, copy number variation-driven eRNA (chr10:3848037–3849536) showed significantly up-regulated expression and copy number amplification in BRCA (Fig. S4B, C), which is located in a genomic region marked by the up-regulated signals of DNase-seq and H3K4me3 signals in MCF7 as compared with normal cell line (Fig. S5A, B). Using H3K27ac ChIP-seq data (GSE190163) treated with CREBBP/EP300 inhibitors, we found a significant decrease in the H3K27ac signal in the eRNA region (chr10:3848037–3849536) (Fig. S5C). A decreased H3K27ac signal was demonstrated to inhibit the growth of breast cancer cells in our previous study.^5^ Previous studies confirmed that down-regulation of the target genes SPP1, COL1A1, and FN1 of the eRNA (chr10:3848037–3849536) can inhibit the proliferation and invasion of cancer cells.

We applied our integrated approach to identify candidate small molecules that are predicted to inhibit the activity of the eRNAs. Five drugs were identified as the candidate small molecules for prognostic eRNA (chr10:3848037–3849536; Fig. 1G). GSEA analysis showed that co-expressed genes of the eRNA tended to be down-regulated after drug perturbation (Fig. 1H; Fig. S5D). Wortmannin was the top-ranked candidate drug for inhibiting the eRNA, with the shortest path length value and the highest prediction score. Wortmannin, a PI3K inhibitor, could decrease the expression of PI3K and its upstream regulator SPP1. We found that wortmannin significantly inhibited the expression of eRNA co-expressed genes COL1A1 (fold change = 0.84) and FN1 (fold change = 0.75) using CMap data (Fig. S5E). Previous studies found that wortmannin was able to inhibit SPP1 expression, thereby inhibiting cell proliferation in a variety of cancers. Tretinoin was the second top-ranked candidate drug for inhibiting eRNA. It could influence the eRNA co-expressed genes LCN1 and GPRC5A, thereby decreasing the expression of LCN1 and GPRC5A (Fig. S5E, F). Valproic acid was the third top-ranked candidate drug for inhibiting eRNA by influencing the interaction between its co-expressed genes COL1A1 and FN1 (Fig. 1I). Based on CMap data, we found that valproic acid could significantly reduce the expression of FN1 (fold change = 0.73; Fig. S5E). The expression of COL1A1 and FN1 genes has been reported to significantly reduce in valproic acid-treated tissues. Co-expressed genes of eRNA (chr10:3848037–3849536), including extracellular matrix components SPP1, COL1A1, and FN1, were significantly up-regulated in BRCA patients (Fig. 1J, K). Extracellular matrix components, receptors, and associated signaling molecules, including FN1, SPP1, COL1A1, ITGA3, ITGA4, ITGA7, ITGB1, ITGB3, AKT1, and PTEN, were significantly dysregulated in BRCA (Fig. 1K). AKT1 was up-regulated and PTEN was down-regulated, and down-regulation of PTEN could favor increased expression of AKT, thereby promoting cell proliferation. To investigate the significance of eRNAs on specific subtypes of breast cancer, we compared the expression profiles of prognostic eRNAs and their target genes among the four breast cancer subtypes (LumA, LumB, HER2, and Basal-like). We observed a subtype-specific expression pattern of prognostic eRNAs in breast cancer. For example, the eRNA (chr10:3848037–3849536) was specifically highly expressed in the HER2-enriched breast subtype and its target genes were significantly enriched in the highly expressed genes of the HER2 subtype (Fig. S6A, B). Moreover, the expression of eRNA (chr10:3848037–3849536) in the HER2 subtype could distinguish high- and low-risk patients with different prognoses (*P* = 0.04; Fig. S6C). Furthermore, we found that wortmannin, risperidone, and rottlerin could significantly inhibit subtype-specific target genes of the eRNA (chr10:3848037–3849536) in all the four breast cancer subtypes, whereas tretinoin and valproic acid were only effective in Basal-like, LumA, and LumB subtypes (Fig. S6D). Wortmannin has been reported to inhibit gene expression in various subtypes of breast cancer, thereby inhibiting proliferation. Altogether, up-regulation of eRNA (chr10:3848037–3849536) could increase the expression of extracellular matrix components and activate the PI3K/Akt signaling transduction, thereby promoting the tumorigenic phenotype. We predicted wortmannin and valproic acid as potential anticancer drugs for blocking the activity of eRNA by targeting the main components of the extracellular matrix, including SPP1, COL1A1, and FN1. Our findings indicate these copy number variation-driven eRNAs could be used as predictive biomarkers or as potential targets in BRCA.

## Author contributions

LW, HYZ, and CYZ designed the study, implemented the algorithm, and performed the analysis. LW, HYZ, CYZ, and LXW wrote and revised the manuscript. YPS, KLL, YL, and MTF helped collect the data and prepared the figures and tables. LB and WYL helped revise the manuscript.

## Conflict of interests

The authors declare no competing interests.

## Funding

This work was supported by the Natural Science Foundation of Heilongjiang Province (China) (LH2023F012); University Excellent Youth Project of Provincial Scientific Research Institute (Heilongjiang, China)(CZKYF2022-1-C006); Outstanding Youth Foundation of Heilongjiang Province (China) (YQ2023F004); Program for Young Scholars with Creative Talents in Heilongjiang Province (China) (UNPYSCT-2020174).
