# Supplementary material for: Comprehensive identification and potential application of genetic alteration-driven enhancer RNAs for eRNA-targeted therapy in breast cancer
Source: Genes Dis. 2023 Sep 23;11(5):101124. doi: 10.1016/j.gendis.2023.101124 (PMC11167238; doi:10.1016/j.gendis.2023.101124)
Supplement: Multimedia component 1 [file mmc1.pdf]

Supplementary Material

Results

Identifying genetic alteration-driven eRNAs in breast cancer

We generated an integrative pipeline to identity genetic alteration-driven eRNAs in BRCA by integrating somatic mutation, DNA methylation, copy-number variation and TCeA (The Cancer eRNA Atlas, https://bioinformatics.mdanderson.org/public-software/tcea) eRNA expression data (Fig. 1)[1]. First, we identified 5441 active eRNAs that were detected with expression across >10% of BRCA samples[2]. The 1159 differentially expressed eRNAs were recognized by comparing tumor samples with adjacent samples using paired t-test with the threshold value of FDR < 0.05 and FC > 2, including 537 upregulated eRNAs and 622 downregulated eRNAs (Fig. S1A, B). Next, we identified differential DNA methylations, somatic mutations, and copy number variations (CNVs) in BRCA (Fig. S1C-E). Finally, genetic alteration-driven eRNAs was identified if at least one of differential methylation sites, somatic mutations or CNVs was located in the differentially expressed eRNAs and if the dysregulated pattern of the genetic alterations corresponded with transcriptional dysregulation. As a result, 153 DNA methylation-driven eRNAs, one mutation-driven eRNAs, and 369 CNV-driven eRNAs were identified (Fig. S1E). We constructed circular maps to obtain a global overview for the basic information and genetic alteration of 431 genetic alteration-driven eRNAs in BRCA (Fig. 1A). Notably, the majority of the eRNAs (78.65%, 339/431) had only one type of genetic alterations (Fig. S1D, E). We found that six genetic alteration-driven eRNAs in BRCA significantly overlapped with known

breast cancer enhancers when analyzed using DiseaseEnhancer (*P*=0.02; hypergeometric test; Fig. 1B)[3]. For example, eRNA (chr20:46700957-46701130) is a known risk factor for BRCA[4] that showed copy number amplification and upregulated expression in breast cancer samples (Fig. 1C). SULF2, a known target of the eRNA (chr20:46700957-46701130), showed significant upregulation (one-way ANOVA, P=1.04e-9) and positive correlation with the eRNA (R=0.45, P=1.11e-62; Fig. S1F, G). Overexpression of SULF2 was reported to promote cell migration and invasion of breast cancer cells[5]. Both of two eRNAs (chr10:9115735-9116358 and chr10:8903701-8904023) were transcribed from the same super-enhancer region[6] and showed copy number amplification and upregulated expression in BRCA samples (Fig. 1C). The eRNAs (chr10:9115735-9116358 and chr10:8903701-8904023) and their target gene GATA3 expression were positively correlated in BRCA (Fig. 1C and Fig. S1F, G). The expression of GATA3 was reported to play a role in controlling the occurrence and metastasis of breast tumor[7] and GATA3 was used to diagnose luminal A and luminal B breast subtypes[8]. Therefore, those genetic alteration-driven eRNAs may serve as potential markers for cancer diagnosis and treatment and be used for subsequent analysis.

Identifying candidate small molecules affecting genetic alteration-driven eRNA activity

Studies showed that eRNAs could induce oncogenes expression and may be considered potential anticancer drug targets[9]. We generated an integrative pipeline to identity candidate small molecules that can affect genetic alteration-driven eRNA

activity (Fig. 1D). First, we identified differentially coexpressed genes of genetic alteration-driven eRNAs as their potential target genes[2, 10]. A total of 69465 eRNA-target pairs involving 259 genetic alteration-driven eRNAs and 3471 differentially expressed genes were identified. Next, the Gene Set Enrichment Analysis (GSEA) approach[11] was implemented to calculate whether target genes of genetic alteration-driven eRNAs are significantly affected by drug perturbations based on CMap[12]. Positive normalized enrichment scores (NES) from GSEA analysis suggested that drug induced the upregulation of eRNA targets, whereas negative scores signified drug-induced downregulation of the targets. Candidate drugs for perturbing the driving role of genetic alteration-driven eRNAs (drug-eRNA pairs) were identified with the threshold of FDR-adjusted P-value <0.05. We obtained 100856 drug-eRNA pairs involving 238 genetic alteration-driven eRNAs and 1252 drugs, among that NES values of 32582 drug-enhancer pairs were greater than 0 and NES values of 68274 pairs were less than 0. Finally, based on a curated biological network containing 28712 drugs, 17553 protein-coding genes and 259 genetic alteration-driven eRNAs, the standardized shortest path length (SPL) between the drug and eRNA was calculated. Regulation index (RI) for each candidate drug-eRNA pair was calculated as a final prediction score by integrating the NES and SPL values. Higher RI values indicated that the drug was a better candidate for perturbing the expression of eRNA. For each eRNA, we obtained the top five drugs as predicted small molecules that affect its expression and functions. In total, there were 1139 predicted drug-eRNA pairs involving 234 eRNAs and 343 drugs (Fig. 1D). There were 443 predicted activating drug-eRNA pairs (a drug induces

activation of eRNA target genes) and 696 inhibiting drug-eRNA pairs (a drug induces repression of eRNA target genes). We found that six predicted small molecules significantly overlapped with known BRCA drugs such as fulvestrant, methotrexate and paclitaxel (*P*=5.3e-3; hypergeometric test; Fig. S2A) and known anti-cancer drugs such as irinotecan and daunorubicin (*P*=1.2e-3; Fig. S2B). Some hub small molecules have been reported to demonstrate potential as anti-cancer agents in BRCA. For example, trichostatin A (TSA) was reported to induce apoptosis of breast cancer cell lines and inhibit proliferation, invasion and migration of breast cancer cells[13]. Tolfenamic acid (TA) can inhibit proliferation and induce apoptosis of MDA-MB-231 breast cancer cells and tumor xenograft models[14]. Mefloquine has also been reported to have anti-cancer effect in breast cancer cell lines[14], and to be synergistic with doxorubicin/paclitaxel for treatment of BRCA[15]. Spiperone has been reported to cause apoptosis of MCF7 cells and is cytotoxic to BRCA[16]. Network analysis showed that the SPL values of the predicted drug-eRNA pairs were significantly shorter than those of nonpredictions (GSEA FDR=1.0) (*P*=1.43e-09, *P*<2.2e-16, Mann-Whitney test; Fig. S2C-E). Most of the SPL values of the predicted drug-eRNA pairs were 2 (Fig. S2C-F), which means that our predictions were not random and that the predicted drugs might disrupt eRNAs by targeting regulatory or interacting cofactors.

For example, a known risk eRNA for breast cancer (chr10:9115735-9116358) showed upregulated expression (paired t-test, FC = 25.6, P=0.01, Fig. S3A, B) and copy number amplification in breast cancer (Fig. S3C). Consistent with this, the eRNA is located in a genomic region marked by upregulated signals of DNase-seq, H3K27ac

and H3K4me3 in MCF7 cell line (Fig. S3A), indicating its potential risk of breast cancer. We identified 5 candidate drugs which were predicted to inhibit eRNA (chr10:9115735-9116358) activity, including isoniazid, chlorogenic acid, hydrastinine, sulmazole, and pheniramine (Fig. S3D, E). GSEA analysis showed that co-expressed genes of eRNA (chr10:9115735-9116358) tended to be downregulated after drug perturbation (Fig. S3E). Isoniazid is identified as the drug with the shortest path length and highest prediction score (Fig. S3D). Isoniazid is reported to be able to act as a potential therapeutic drug for prostate cancer treatment[17] and its derivative ITHB4 demonstrated the ability to inhibit the growth of MCF-7 breast cancer cells[18]. We found that isoniazid could decrease the expression of eRNA (chr10:9115735-9116358) through disruption of its co-expressed gene CYP2B6 in breast cancer cells. CYP2B6, a positively co-expressed gene of the eRNA, showed significantly upregulated expression in breast cancer cells and was reversed by isoniazid in the CMap profile (FC=0.72; Fig. S3A; Fig. S3F, G). CYP2B6 is a known estrogen receptor (ER) target gene that has been previously implicated in testosterone metabolism and breast cancer risk[19, 20]. Chlorogenic acid is the top 2 ranked candidate drug for inhibiting eRNA (chr10:9115735-9116358) expression through influencing the interaction of EHMT2, ESR1, FOXA1 and GATA3 (Fig. S3D). Histone methyltransferase EHMT2 is the target gene of chlorogenic acid[21] and interacts with ESR1 in HINT database[22]. ESR1 and its interacting cofactors FOXA1 and GATA3 are positively co-expressed genes of the eRNA which showed upregulated expressed in breast cancer (Fig. S3G, H).

Genetic alteration-driven eRNAs acting as prognostic markers of breast cancer

To characterize the prognostic value of genetic alteration-driven eRNAs in BRCA, we analyzed the expression of 431 genetic alteration-driven eRNAs in 1094 patients. The patient demographic characteristics were shown in Table S1. The median follow-up time for the cohort was 5.6 months and 340 out of 1094 patients (74.7%) were alive at the time of the last follow-up. Tumor staging data revealed that 804, 269 and 13 patients belonged to stages 1-2, 3-4, and X, respectively. American Joint Committee on Cancer (AJCC) pathologic tumor (pT) staging showed that 916, 178 and 3 BRCA patients belonged to T1-T2, T3-T4 and TX groups. AJCC pathologic metastasis (pM) staging showed that 912, 22, and 163 patients belonged to M0, M1 and MX groups. Univariate Cox proportional hazards analysis showed that age, HER2 status, TNM stage, AJCC pathological tumor stage and six genetic alteration-driven eRNAs (chr10:3848037-3849536, chr10:5530637-5531333, chr6:118759105-118759218, chr8:116990613-116990894, chr8:117175146-117175377 and chr8:117235182-117235423) were significantly associated with overall survival (Table S2). We performed multivariate Cox proportional hazards model analysis on the expression of the six eRNAs in relation to clinical parameters such as age at diagnosis, gender, ER status, PR status, HER2 status, TNM stage and AJCC pathologic tumor stage. We identified three eRNAs, including chr10:3848037-3849536 (HR=3.00, 95% CI: 1.18 to 7.66, P=0.02), chr8:116990613-116990894 (HR=5.26, 95% CI: 1.54 to 17.96, P=0.008) and chr6:118759105-118759218 (HR=0.04, 95% CI: 0.002 to 0.89, P=0.04), as independent risk factors for BRCA prognosis (Fig. S4A; Table S3). We found eRNA (chr8:116990613-116990894) transcribed from an intronic region of the long non-

coding RNA LINC00536 that had been reported to predict overall survival for patients with BRCA[23]. The Kaplan–Meier survival curve showed that higher expression of two eRNAs (chr10:3848037-3849536 and chr8:116990613-116990894) and lower expression of one eRNA (chr6:118759105-118759218) were associated with poorer prognosis (log-rank test, P=0.04, P=0.02 and P=0.03, respectively; Fig. 1E). Furthermore, we characterized the prognostic value of CNVs of eRNAs. We identified two eRNAs (chr10:3848037-3849536 and chr8:116990613-116990894) whose copy number amplifications were significantly associated with a poor prognosis (log-rank test, P=0.019 and P=0.020, respectively; Fig. 1F). For example, CNV-driven eRNA (chr10:3848037-3849536) showed significantly upregulated expression (paired t-test, FC=5.83, P=1.0e-7) and copy number amplification in BRCA (Fig. S4B, C) which is located in a genomic region marked by upregulated signals of DNase-seq and H3K4me3 signals in MCF7 as compared with normal cell line (Fig. S5A). We found an association between the prognostic eRNA and clinical parameters, such as HER2 status, M stage and T stage (Kruskal-Wallis test, P<0.05, Fig. S5B). Using H3K27ac ChIP-seq data (GSE190163) treated with CREBBP/EP300 inhibitors, we found a significant decrease in the H3K27ac signal in the eRNA region (chr10:3848037-3849536) (Fig. S5C). A decreased H3K27ac signal was demonstrated to inhibit the growth of breast cancer cells in our previous study. Previous studies confirmed that downregulation of the target gene SPP1, COL1A1, and FN1 of the eRNA (chr10:3848037-3849536) can inhibit the proliferation and invasion of cancer cells.

We applied our integrated approach to identify candidate small molecules that are predicted to inhibit the activity of the eRNAs (Fig. 1G; Fig. S5D–F). As a result, five drugs were identified as the candidate small molecules for prognostic eRNA (chr10:3848037-3849536) (Fig. 1G). GSEA analysis showed that coexpressed genes of the eRNA tended to be downregulated after drug perturbation (Fig. 1H; Fig. S5D). Wortmannin was the top-ranked candidate drug for inhibiting the eRNA, with the shortest SPL value and the highest prediction score (Fig. 1G). It could decrease the expression of eRNA through disruption of its coexpressed gene SPP1. Wortmannin, a PI3K inhibitor, could decrease the expression of PI3K and its upstream regulator SPP1 using CMap data. We found that wortmannin significantly inhibited the expression of eRNA coexpressed genes COL1A1 (FC = 0.84) and FN1 (FC = 0.75) using CMap data (Fig. S5E). Previous studies found that wortmannin was able to inhibit SPP1 expression, thereby inhibiting cell proliferation in a variety of cancers. Tretinoin was the second top-ranked candidate drug for inhibiting eRNA. It could influence the eRNA coexpressed genes LCN1 and GPRC5A, thereby decreasing the expression of LCN1 (FC= 0.74) and GPRC5A (FC= 0.47) in breast cancer cells using CMap data (Fig. S5E, F). Valproic acid was the third top-ranked candidate drug for inhibiting eRNA through influencing the interaction between its coexpressed gene COL1A1 and FN1 (FC= 0.75; Fig. 1I). Valproic acid was a histone deacetylase inhibitor and was reported to induce apoptosis of breast cancer stem cells[24]. Based on CMap data, we found that valproic acid could significantly reduce the expression of FN1 (FC = 0.73; Fig. S5E). The expression of COL1A1 and FN1 genes has been reported to significantly reduce in

valproic acid-treated tissues. Coexpressed genes of eRNA (chr10:3848037-3849536), including extracellular matrix (ECM) components SPP1, COL1A1, and FN1, were significantly upregulated in breast cancer patients (Fig. 1J, K). These altered genes were significantly associated with the pathways of ECM-receptor interaction (FDR=2.2e-3), focal adhesion (FDR=5.7e-3) and PI3K-Akt signaling pathway (FDR=8.9e-3), according to DAVID[25]. ECM components, receptors, and associated signaling molecules, including FN1, SPP1, COL1A1, ITGA3, ITGA4, ITGA7, ITGB1, ITGB3, AKT1 and PTEN, were significantly dysregulated in BRCA (Fig. 1K). Increasing evidence had shown that overexpressed ECM components such as SPP1, COL1A1 and FN1 were related to breast cancer cell proliferation and invasion[26, 27]. AKT serine/threonine kinase 1 (AKT1) was upregulated and phosphatase and tensin homolog (PTEN) was downregulated, and downregulation of PTEN could favor increased expression of AKT, thereby promoting the cell proliferation[28]. To investigate the significance of eRNAs on specific subtypes of breast cancer, we compared the expression profiles of prognostic eRNAs and their target genes among the four breast cancer subtypes (LumA, LumB, HER2, and Basal-like). We observed a subtype-specific expression pattern of prognostic eRNAs in the breast cancer. For example, the eRNA (chr10:3848037-3849536) was specifically highly expressed in the HER2-enriched breast subtype and its target genes were significantly enriched in the highly expressed genes of HER2 subtype (Fig. S6A and B). Moreover, the expression of eRNA (chr10:3848037-3849536) in the HER2 subtype could distinguish high- and low-risk patients with different prognosis (P = 0.04; Fig. S6C). Furthermore, we found

that wortmannin, risperidone and rottlerin could significantly inhibit subtype-specific target genes of the eRNA (chr10:3848037-3849536) in all of the four breast cancer subtypes, whereas tretinoin and valproic acid were only effective in Basal-like, LumA and LumB subtypes (Fig. S6D). Wortmannin had been reported to inhibit gene expression in various subtypes of BRCA, thereby inhibiting proliferation. Altogether, upregulation of eRNA (chr10:3848037-3849536) could increase the expression of ECM components and activate the PI3K/Akt signaling transduction, thereby promoting the tumorigenic phenotype. We predicted wortmannin and valproic acid as potential anticancer drugs for blocking the activity of eRNA by targeting the main components of ECM, including SPP1, COL1A1 and FN1. Our findings indicate these CNV-driven eRNAs could be used as predictive biomarkers or as potential targets in BRCA.

Materials and Methods

Datasets

We downloaded the expression profile of eRNA from the The Cancer eRNA Atlas (TCeA) data portal[29] and the RNA-seq data of breast cancer from The Cancer Genome Atlas (TCGA). Copy number aberrations of breast cancer were obtained from GDAC database, while the HM450k array datasets and the Whole Genome Bisulfite Sequencing (WGBS) datasets were collected from TCGA. Mutations and the corresponding clinical metadata of breast cancer was accessed from the public cBio Cancer Genomics Portal. The protein-protein interactions were collected from the Homologous Interactions (HINT) database[30]. The drug-protein intersections were

downloaded from DrugBank[31] and the drug-gene interaction database (DGIdb)[32]. The patients' demographic characteristics are shown in Table S1.

Comparison of eRNA activation and gene activation between tumor and normal samples in BRCA

We first obtained RNA-seq data and the expression profile of eRNAs of breast cancer from the TCGA and FANTOM5 database[33]. Then, an eRNA was considered as expressed in breast cancer if observed in >10% of the samples. We identified 5441 active eRNAs in breast cancer. The difference of each eRNA expression between 113 breast cancer samples and matched adjacent normal samples was evaluated. Log2 transformed fold change was calculated to quantify the expression change from tumor to normal samples. Statistics were performed using paired t-test. The eRNAs with absolute fold change > 2 and the Benjamini-Hochberg false discovery rate (FDR-BH) adjusted P-value < 0.05 were defined as differentially expressed eRNAs. For RNA-seq data, differentially expressed genes were identified using DESeq2[34].

Annotation of genetic alterations in eRNAs

We obtained mutation data, copy number data and DNA methylation data of TCGA breast cancer. For the mutated eRNAs analysis, we integrated mutation data with eRNA region data, then used the bedtools tool[35] to find the overlap between the mutation site and the eRNA region, the mutated eRNA was defined as if the mutation appears in the eRNA region. For the copy-number alteration eRNAs analysis, we first identified regions of copy number variation using the GISTIC2.0 software with q<0.25[36], then identified the overlap between the CNV region and the eRNA region using the bedtools

tool, the copy-number alteration eRNA was defined as if the CNV region appears in the eRNA region. For the differentially CpG methylated eRNAs analysis, we first identified regions of differentially CpG methylation using the LncDM R package (T-test, FDR<0.05)[37], then identified the overlap between the differentially CpG methylated site and the eRNA region using the bedtools tool, the differentially CpG methylated eRNA was defined as if the differentially CpG methylated site appears in the eRNA region. The reference genome for all data was hg19.

Derivation of eRNA co-expression target gene sets

We used co-expression analysis to identify the target genes of eRNAs. For each genetic alteration-driven eRNA, the expression correlations between each eRNA and differentially expressed genes were calculated using Spearman's rank correlation, and the FDR-BH adjusted P-value were calculated. Genes were considered as targets of a eRNA if Spearman's rho > 0.3 and FDR < 10e-4[29, 38]. This analysis produced a direct target gene set for each genetic alteration-driven eRNA.

Connectivity Map

The Connectivity Map is a database containing genome-wide transcription data after treatment of cultured cells with different types of reagents (mainly small molecule compounds)[39], and after treatment of cultured cells with the vehicle controls. The cultured human cell lines were MCF7, HL60, SKMEL5, and PC3 NCI60 cell lines, and the number of compounds was 1309. In order to explore the relationship between the drug and its target, we obtained more than 7000 expression profile from the CMap database for 1309 compounds and vector controls experiments. For each compound,

the differential expression profiles were calculated as the mean level of difference between drug and vector controls across all experiments and cell lines, then we generated a ranked gene lists by sorting the differential gene expression profiles from the most up-regulated to the most down-regulated genes.

Identification of candidate drugs affecting genetic alteration-driven eRNA

To identify candidate small molecules that affect genetic alteration-driven eRNA, we first identified genetic alteration-driven eRNA co-expression target gene sets, and generated ranked gene lists from drug-induced gene expression profiles based on the Broad Institute's Connectivity Map. We then calculated whether drug perturbations could significantly affect target genes of genetic alteration-driven eRNAs by performing GSEA for each drug in the Connectivity Map. We consider a drug to be predicted to affect eRNA activity if the FDR adjusted p-value is less than 5% (FDR<0.05) and the normalized enrichment score is used as a score that indicates the strength of the prediction. Finally, we computed the shortest path length and standardized path length between all drug-eRNA pairs in the biological network. The standardized path length was computed as follows:

$$\mathrm{SPL} = \mathrm{P}(\mathrm{PL}|\mathrm{d},\mathrm{e})\,\frac{\sum_{\mathrm{i}=1}^{500} PL_{d,e,xi} < PL_{d,e}}{500}$$

where d represents drugs, e represents eRNAs, $PL_{d,e,xi}$ represents the shortest path length observed in random networks x_i and $PL_{d,e}$ represents the shortest path length observed in the observed network. The final prediction score called the regulation index (RI) by integrating the result of GSEA (NES) and the network standardized path length (SPL). The regulation index was computed as follows:

$$RI_{d,e} = \frac{NES_{d,e}}{SPL}$$

where d represents drugs, e represents eRNAs, $NES_{d,e}$ represents the normalized enrichment score for a drug-eRNA pair and SPL represents the standardized path length between the drug and eRNA in the biological network.

Survival analysis

To evaluate the prognosis effect of genetic alteration-driven eRNAs, we obtained corresponding clinical metadata of breast cancer from the public cBio Cancer Genomics Portal. We first assessed the effect of all genetic alteration-driven eRNAs and clinical factors alone on overall survival using univariate Cox regression model and log-rank test and considered $p < 0.05$ as significant, then the multivariate Cox proportional hazards regression model was performed with remaining eRNAs and clinical parameters (medage, sex, IHC_ER, IHC_HER2, IHC_PR, AJCC_M, AJCC_N, AJCC_T, AJCC_STAGE), where crude hazard ratios (HRs) and 95% confidence intervals (CIs) were calculated, and eRNAs with $p < 0.05$ were considered as prognostic eRNAs. The Kaplan-Meier (K-M) method was used to create survival plots and log-rank test was used to compare the difference of survival curves.

References

1. Chen, H. and H. Liang, *A High-Resolution Map of Human Enhancer RNA Loci Characterizes Super-enhancer Activities in Cancer.* Cancer Cell, 2020. **38**(5): p. 701-715 e5.
2. Chen, H., C. Li, X. Peng, Z. Zhou, J.N. Weinstein, N. Cancer Genome Atlas Research, and H. Liang, *A Pan-Cancer Analysis of Enhancer Expression in Nearly 9000 Patient Samples.* Cell, 2018. **173**(2): p. 386-399 e12.
3. Zhang, G., J. Shi, S. Zhu, Y. Lan, L. Xu, H. Yuan, G. Liao, X. Liu, Y. Zhang, Y. Xiao, et al., *DiseaseEnhancer: a resource of human disease-associated enhancer catalog.* Nucleic Acids Res, 2018. **46**(D1): p. D78-D84.

4. Kim, K., K. Jang, W. Yang, E.Y. Choi, S.M. Park, M. Bae, Y.J. Kim, and J.K. Choi, *Chromatin structure-based prediction of recurrent noncoding mutations in cancer.* Nat Genet, 2016. **48**(11): p. 1321-1326.

5. Viera, M., G.W.C. Yip, H.M. Shen, G.H. Baeg, and B.H. Bay, *Targeting CD82/KAI1 for Precision Therapeutics in Surmounting Metastatic Potential in Breast Cancer.* Cancers (Basel), 2021. **13**(17).

6. Gao, T. and J. Qian, *EnhancerAtlas 2.0: an updated resource with enhancer annotation in 586 tissue/cell types across nine species.* Nucleic Acids Res, 2020. **48**(D1): p. D58-D64.

7. Bai, F., L.H. Zhang, X. Liu, C. Wang, C. Zheng, J. Sun, M. Li, W.G. Zhu, and X.H. Pei, *GATA3 functions downstream of BRCA1 to suppress EMT in breast cancer.* Theranostics, 2021. **11**(17): p. 8218-8233.

8. Perou, C.M. and A.L. Borresen-Dale, *Systems biology and genomics of breast cancer.* Cold Spring Harb Perspect Biol, 2011. **3**(2).

9. Napoli, S., N. Munz, F. Guidetti, and F. Bertoni, *Enhancer RNAs (eRNAs) in Cancer: The Jacks of All Trades.* Cancers (Basel), 2022. **14**(8).

10. Fishilevich, S., R. Nudel, N. Rappaport, R. Hadar, I. Plaschkes, T. Iny Stein, N. Rosen, A. Kohn, M. Twik, M. Safran, et al., *GeneHancer: genome-wide integration of enhancers and target genes in GeneCards.* Database (Oxford), 2017. **2017**.

11. Lamb, J., E.D. Crawford, D. Peck, J.W. Modell, I.C. Blat, M.J. Wrobel, J. Lerner, J.P. Brunet, A. Subramanian, K.N. Ross, et al., *The Connectivity Map: using gene-expression signatures to connect small molecules, genes, and disease.* Science, 2006. **313**(5795): p. 1929-35.

12. Subramanian, A., P. Tamayo, V.K. Mootha, S. Mukherjee, B.L. Ebert, M.A. Gillette, A. Paulovich, S.L. Pomeroy, T.R. Golub, E.S. Lander, et al., *Gene set enrichment analysis: a knowledge-based approach for interpreting genome-wide expression profiles.* Proc Natl Acad Sci U S A, 2005. **102**(43): p. 15545-50.

13. Song, X., J.Q. Wu, X.F. Yu, X.S. Yang, and Y. Yang, *Trichostatin A inhibits proliferation of triple negative breast cancer cells by inducing cell cycle arrest and apoptosis.* Neoplasma, 2018. **65**(6): p. 898-906.

14. Kim, H.J., S.D. Cho, J. Kim, S.J. Kim, C. Choi, J.S. Kim, J.S. Nam, K. Han Kwon, K.S. Kang, and J.Y. Jung, *Apoptotic effect of tolfenamic acid on MDA-MB-231 breast cancer cells and xenograft tumors.* J Clin Biochem Nutr, 2013. **53**(1): p. 21-6.

15. Duarte, D. and N. Vale, *New Trends for Antimalarial Drugs: Synergism between Antineoplastics and Antimalarials on Breast Cancer Cells.* Biomolecules, 2020. **10**(12).

16. Varalda, M., A. Antona, V. Bettio, K. Roy, A. Vachamaram, V. Yellenki, A. Massarotti, G. Baldanzi, and D. Capello, *Psychotropic Drugs Show Anticancer Activity by Disrupting Mitochondrial and Lysosomal Function.* Front Oncol, 2020. **10**: p. 562196.

17. Yang, X.G., Y.F. Li, Y.J. Wang, R.H. Kong, and D. Wang, *Application of novel pH sensitive isoniazid-heptamethine carbocyanine dye conjugates against prostate cancer cells.* Pharmazie, 2020. **75**(9): p. 412-416.

18. Barathan, M., A.K. Zulpa, K.M. Vellasamy, V. Mariappan, N.K.H. Shivashekaregowda, Z.A. Ibrahim, and J. Vadivelu, *Cytotoxic Activity of Isoniazid Derivative in Human Breast Cancer Cells.* In Vivo, 2021. **35**(5): p. 2675-2685.

19. Lo, R., L. Burgoon, L. Macpherson, S. Ahmed, and J. Matthews, *Estrogen receptor-dependent regulation of CYP2B6 in human breast cancer cells.* Biochim Biophys Acta,

2010. **1799**(5-6): p. 469-79.

20. Justenhoven, C., D. Pentimalli, S. Rabstein, V. Harth, A. Lotz, B. Pesch, T. Bruning, T. Dork, P. Schurmann, N. Bogdanova, et al., *CYP2B6*6 is associated with increased breast cancer risk.* Int J Cancer, 2014. **134**(2): p. 426-30.

21. Tanoli, Z., Z. Alam, M. Vaha-Koskela, B. Ravikumar, A. Malyutina, A. Jaiswal, J. Tang, K. Wennerberg, and T. Aittokallio, *Drug Target Commons 2.0: a community platform for systematic analysis of drug-target interaction profiles.* Database (Oxford), 2018. **2018**: p. 1-13.

22. Patil, A. and H. Nakamura, *HINT: a database of annotated protein-protein interactions and their homologs.* Biophysics (Nagoya-shi), 2005. **1**: p. 21-24.

23. Fan, C.N., L. Ma, and N. Liu, *Systematic analysis of lncRNA-miRNA-mRNA competing endogenous RNA network identifies four-lncRNA signature as a prognostic biomarker for breast cancer.* J Transl Med, 2018. **16**(1): p. 264.

24. Aztopal, N., M. Erkisa, E. Erturk, E. Ulukaya, A.H. Tokullugil, and F. Ari, *Valproic acid, a histone deacetylase inhibitor, induces apoptosis in breast cancer stem cells.* Chem Biol Interact, 2018. **280**: p. 51-58.

25. Dennis, G., Jr., B.T. Sherman, D.A. Hosack, J. Yang, W. Gao, H.C. Lane, and R.A. Lempicki, *DAVID: Database for Annotation, Visualization, and Integrated Discovery.* Genome Biol, 2003. **4**(5): p. P3.

26. Wang, Y., H. Xu, B. Zhu, Z. Qiu, and Z. Lin, *Systematic identification of the key candidate genes in breast cancer stroma.* Cell Mol Biol Lett, 2018. **23**: p. 44.

27. Sponziello, M., F. Rosignolo, M. Celano, V. Maggisano, V. Pecce, R.F. De Rose, G.E. Lombardo, C. Durante, S. Filetti, G. Damante, et al., *Fibronectin-1 expression is increased in aggressive thyroid cancer and favors the migration and invasion of cancer cells.* Mol Cell Endocrinol, 2016. **431**: p. 123-32.

28. Lu, W., S. Jianglun, L. Ning, Z. Yang, H. Feng, D. Hao, C. Haifeng, and Y. Jinyin, *Curcumin Affects Parkinson Protein 7 (PARK7; DJ-1) Expression and Regulates Proliferation and Apoptosis of Breast Cancer Cells by Up-Regulating miR-203 %J JOURNAL OF BIOMATERIALS AND TISSUE ENGINEERING.* 2021. **11**(12).

29. Chen, H. and H. Liang, *A High-Resolution Map of Human Enhancer RNA Loci Characterizes Super-enhancer Activities in Cancer.* Cancer Cell, 2020. **38**(5): p. 701-715 e5.

30. Patil, A. and H. Nakamura, *HINT: a database of annotated protein-protein interactions and their homologs.* Biophysics (Nagoya-shi), 2005. **1**: p. 21-24.

31. Wishart, D.S., et al., *DrugBank 5.0: a major update to the DrugBank database for 2018.* Nucleic Acids Res, 2018. **46**(D1): p. D1074-D1082.

32. Cotto, K.C., et al., *DGIdb 3.0: a redesign and expansion of the drug-gene interaction database.* Nucleic Acids Res, 2018. **46**(D1): p. D1068-D1073.

33. Consortium, F., et al., *A promoter-level mammalian expression atlas.* Nature, 2014. **507**(7493): p. 462-70.

34. Love, M.I., W. Huber, and S. Anders, *Moderated estimation of fold change and dispersion for RNA-seq data with DESeq2.* Genome Biol, 2014. **15**(12): p. 550.

35. Quinlan, A.R. and I.M. Hall, *BEDTools: a flexible suite of utilities for comparing genomic features.* Bioinformatics, 2010. **26**(6): p. 841-2.

36. Mermel, C.H., et al., *GISTIC2.0 facilitates sensitive and confident localization of the targets of focal somatic copy-number alteration in human cancers.* Genome Biol, 2011. **12**(4): p. R41.
37. Zhi, H., et al., *Lnc2Meth: a manually curated database of regulatory relationships between long non-coding RNAs and DNA methylation associated with human disease.* Nucleic Acids Res, 2018. **46**(D1): p. D133-D138.
38. Chen, H., et al., *A Pan-Cancer Analysis of Enhancer Expression in Nearly 9000 Patient Samples.* Cell, 2018. **173**(2): p. 386-399 e12.
39. Lamb, J., et al., *The Connectivity Map: using gene-expression signatures to connect small molecules, genes, and disease.* Science, 2006. **313**(5795): p. 1929-35.

Figure legends and tables

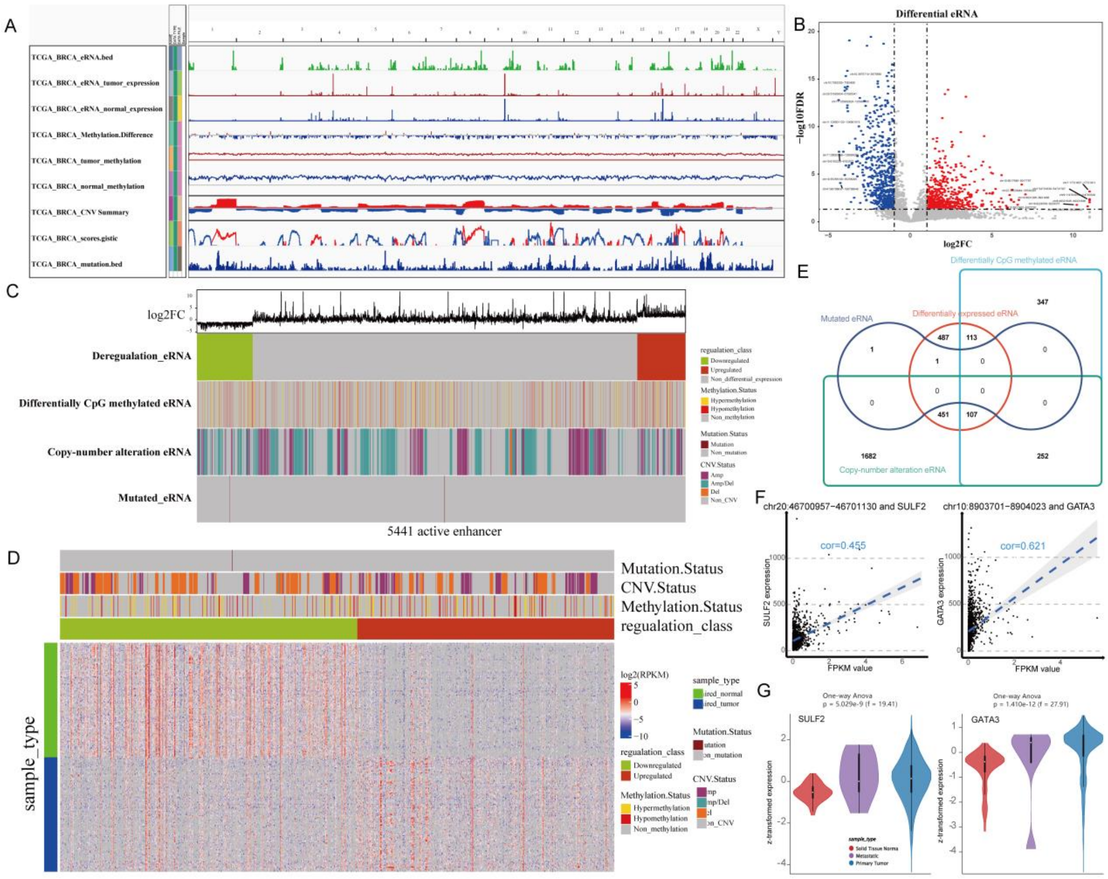

Supplementary Fig. S1. Genetic alterations-driven eRNAs in breast cancer. (A) An overview of 5441 active eRNAs, differential methylation sites, mutations, and copy number variation regions in the whole genome region using the Integrative Genomics Viewer (IGV) tool. (B) The volcano figure of differentially expressed eRNAs in tumor and adjacent samples. (C) The genetic distribution of all active enhancers. (D) Heatmap representation of 1159 differential expression eRNAs' expression in tumor and adjacent samples and genetic variation eRNAs. (E) Venn diagrams showing the overlaps between differential expression eRNAs and three genetic variation eRNAs (CpG methylated eRNAs, Copy-number alteration eRNAs, mutated eRNAs). (F) Expression correlation between eRNAs (chr10:9115735-9116358 and chr10:8903701-8904023)

and their target genes (SULF2 and GATA3) using the Spearman's correlation test. (G) Differential expression of eRNA target genes (SULF2 and GATA3).

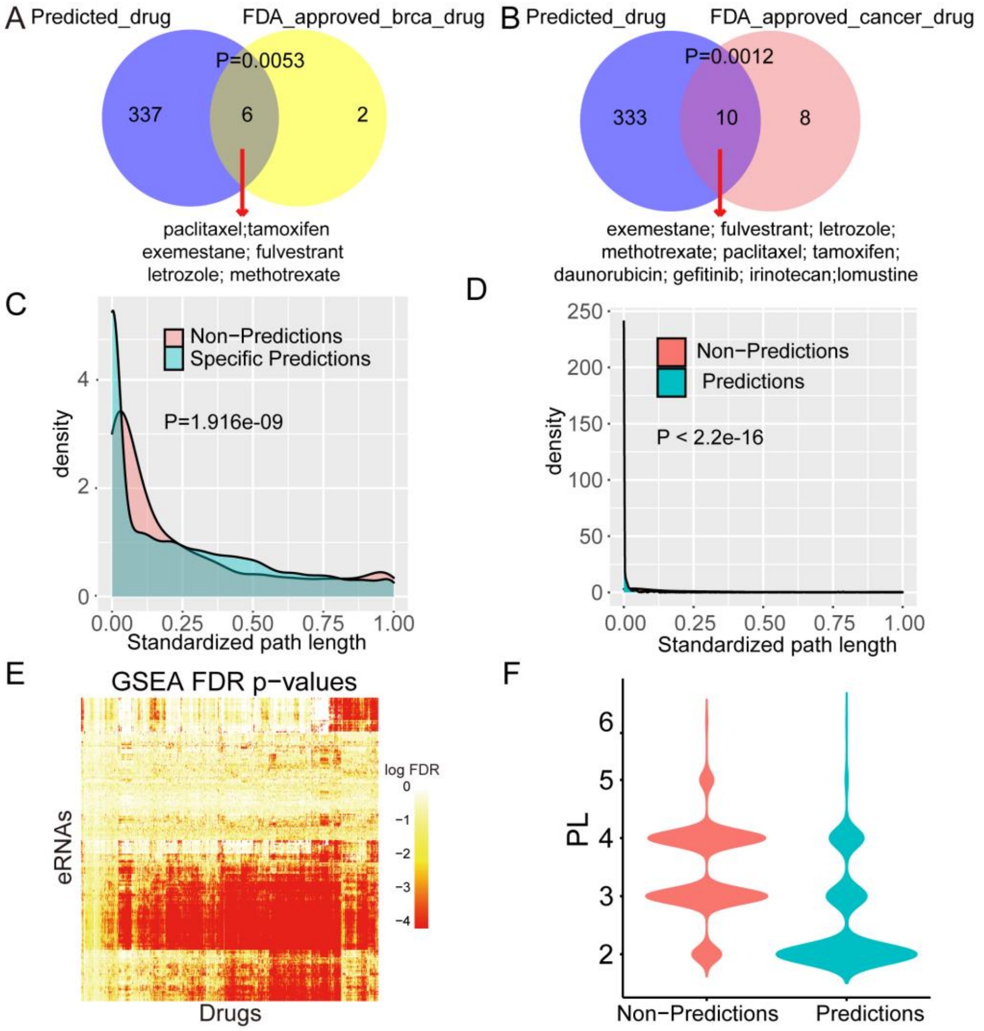

Supplementary Fig. S2. Identifying candidate small molecules affecting genetic alteration-driven eRNA. (A) Intersection of the predicted drugs and the FDA-approved drug of treatment breast cancer. Statistical significance was evaluated using the Hypergeometric distribution test. (B) Intersection of the predicted drugs and the FDA-approved drug of treatment cancer. Statistical significance was evaluated using the Hypergeometric distribution test. (C) Standardized path lengths for the specific predictions and non-predictions (FDR=1). Statistical significance was evaluated using the Mann-Whitney test. (D) Standardized path lengths for the predictions (FDR < 0.05)

and non-predictions (FDR =1). Statistical significance was evaluated using the Mann-Whitney test. (E) Heatmap of the FDR values for genetic alteration driven eRNAs-drug pairs. (F) Distribution of network path lengths (PL) for specific predicted and non-predicted drug-eRNA pairs.

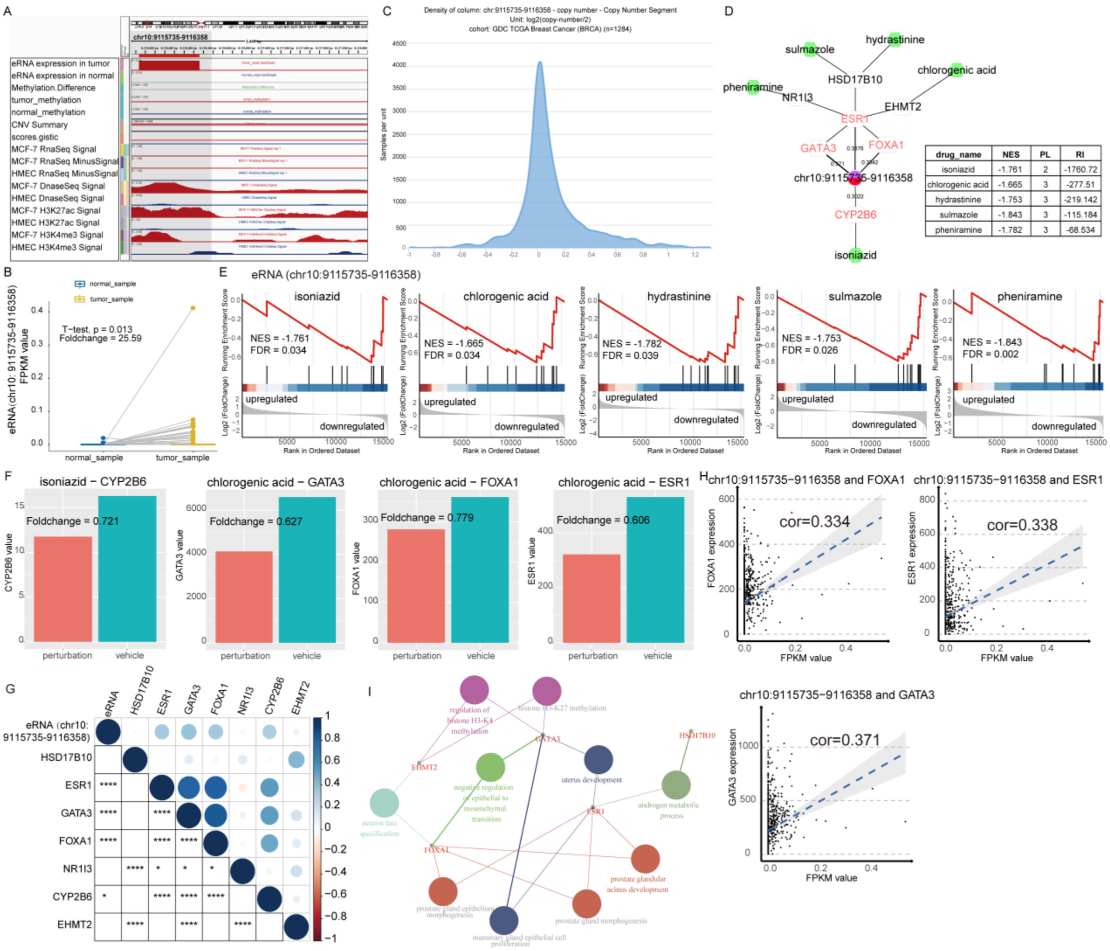

Supplementary Figure S3. Candidate drugs for inhibition of genetic alteration-driven eRNA (chr10:9115735-9116358) in breast cancer. (A) Genetic alterations of eRNA (chr10:9115735-9116358) in TCGA breast cancer samples and histone modifications in MCF-7 and HEMC cell line using the Integrative Genomics Viewer (IGV) tool. The gray shaded areas represents the genomic region of the eRNA. (B) Differential expression of eRNAs (chr10:9115735-9116358) between tumor samples and normal samples. (C) The distribution of copy number segment in the region of

eRNA (chr10:9115735-9116358) in the UCSC database. (D) Network visualization illustrating path lengths from chr10:9115735-9116358 to five candidate drugs. The corresponding table shows metrics that describe each of these drugs in relation to chr10:9115735-9116358. (E) The results of the GSEA for chr10:9115735-9116358 and 5 candidate drugs. The lowest plot shows the log2 differential expression profile for each candidate drug, with the eRNA co-expression target genes marked directly above. Above are the running enrichment scores which illustrate whether the gene set is enriched in the under- or overexpression regions. (F) eRNA-related genes showed differential expression following exposure of breast cancer cells to predicted potential drugs using CMap profile. (G) Expression correlations between the eRNA and target genes. (H) Expression correlation between eRNAs and their target genes (FOXA1, ESR1 and GATA3). (I) The enrichment result of these genes (FOXA1, ESR1, GATA3, EHMT2, CYP2B6, NR1I3 and HSD17B10) in the biological processes.

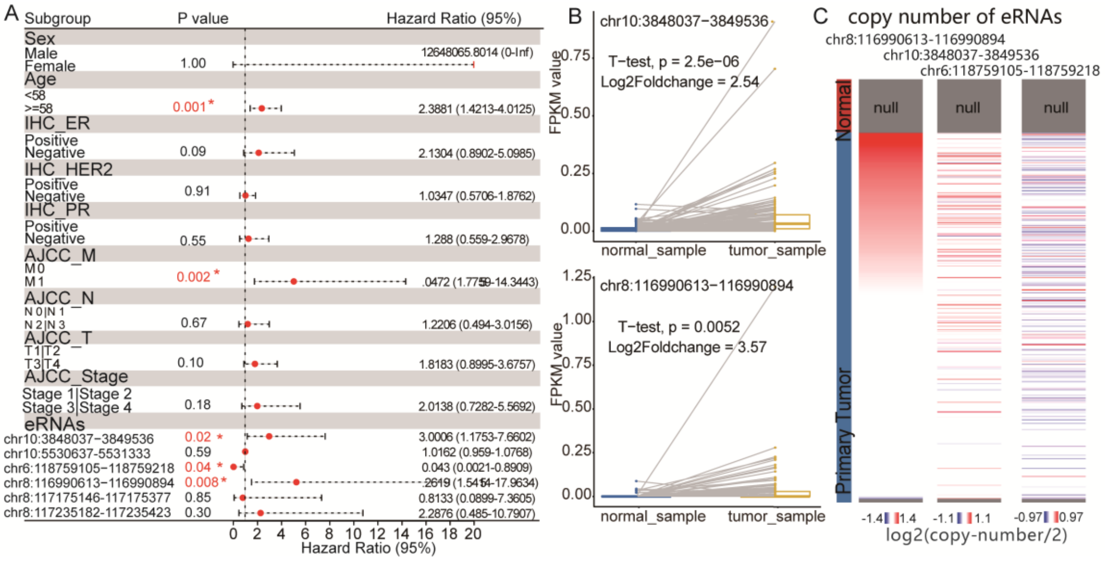

Supplementary Figure S4. Prognosis-related genetic alteration-driven eRNAs in breast cancer. (A) Network visualization illustrating path lengths from the eRNA to

five candidate drugs for eRNA inhibition. Colors of text represent differential expression with red for up-regulation and green for down-regulation. The thickness of the edge represents the correlation coefficient between the two vertices. The fill color of the node represents the genetic alteration of eRNAs. The corresponding table shows metrics that describe each of these drugs in relation to eRNA. NES values are obtained from GSEA, PL is the shortest network path length required to connect eRNA to the drug, and RI is the prediction score by integrating NES values and standardized shortest path lengths. (B) The results for the GSEA involving eRNAs and candidate drugs. (C) Expression correlation between eRNAs and their target genes.

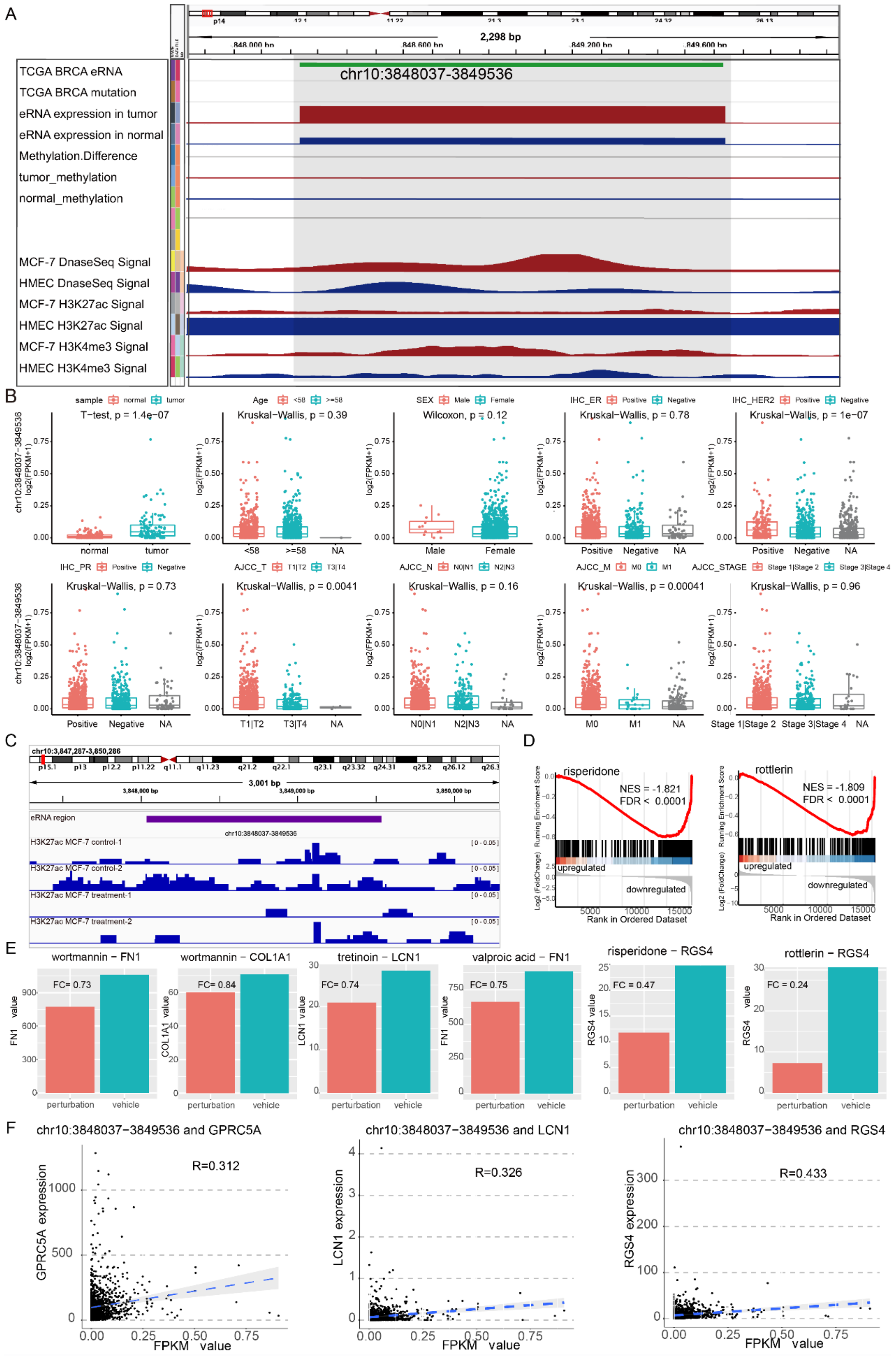

Supplementary Fig. S5. Prognosis-related genetic alteration-driven eRNAs acting as potential drug targets in breast cancer. (A) Genetic alterations of the eRNA

(chr10:3848037-3849536) in TCGA breast cancer samples and histone modifications in MCF-7 and HEMC cell line using the Integrative Genomics Viewer (IGV) tool. The gray shaded areas represents the genomic region of the eRNA. (B) The expression level of the eRNA (chr10:3848037-3849536) was analyzed according to different clinical factors including sample, medage, sex, ER, HER2, PR, T classification, N classification, M classification and AJCC_stage. (C) Histone modification profile of eRNA (chr10:3848037-3849536). Histone modification data were visualized using the Integrative Genomics Viewer (IGV) tool. The control group represents MCF-7 cell line treated with DMSO solution, and the treatment group represents MCF-7 cell line treated with CREBBP/EP300 inhibitors (CPI-1612). (D) The results for the GSEA involving eRNAs and candidate drugs. (E) eRNA target genes showed differential expression following exposure of breast cancer cells to predicted potential drugs using CMap profile. (F) Expression correlation between eRNAs and their target genes.

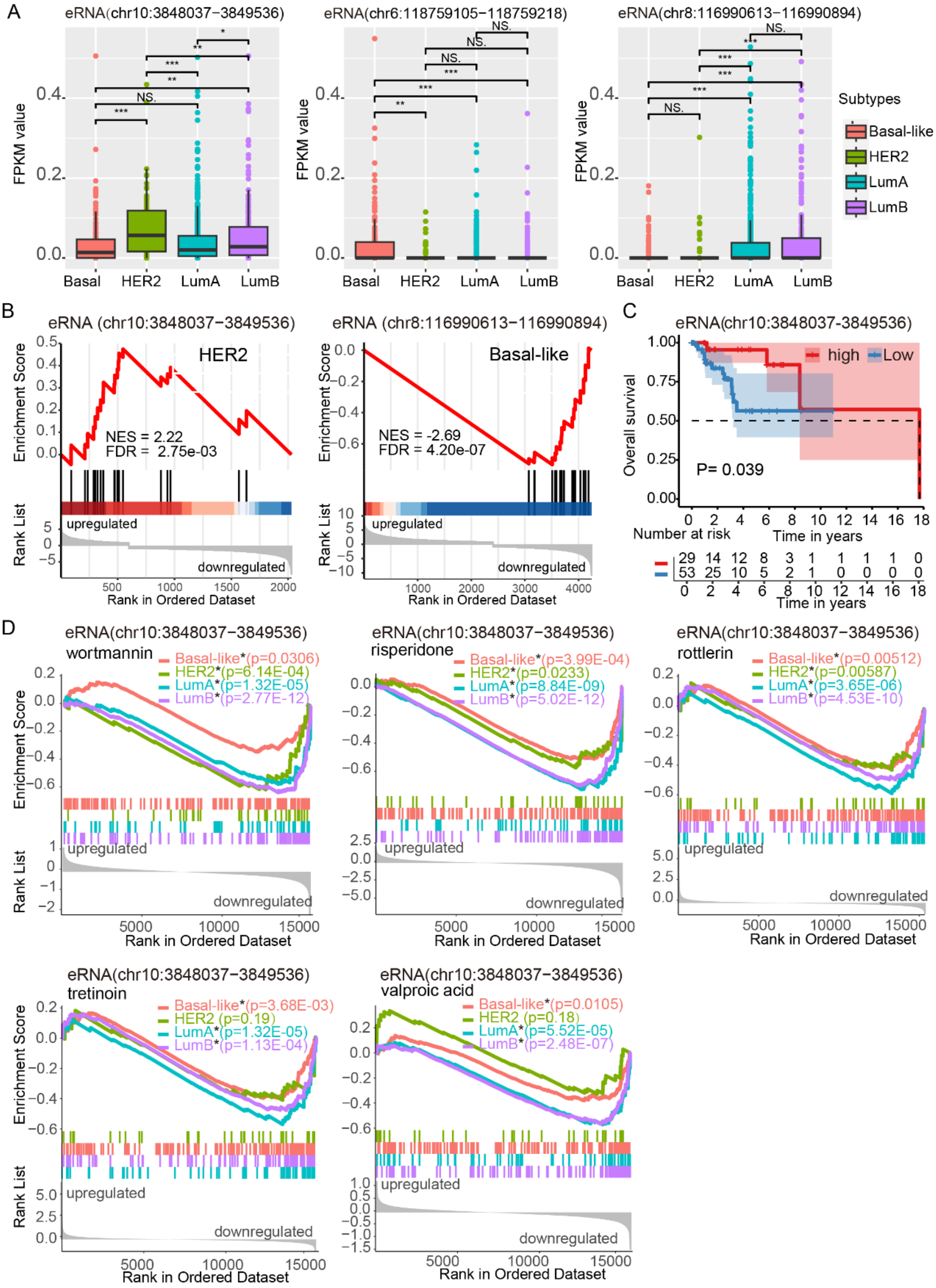

Supplementary Fig. S6. The significance of eRNAs on specific subtypes of breast cancer. (A) The box plot shows the differential expression of genetic alterations-driven eRNA in breast cancer subtypes. Significance indicated by the asterisks (Wilcoxon test, * P< 0.05; ** P < 0.01; *** P < 0.001). (B) The Enrichment map showed that eRNA

target genes were enriched in subtype-specific differentially expressed gene sets. (C) The Kaplan–Meier survival curve based on the expression of eRNA (chr10:3848037-3849536) in the HER2 subtype. (D) The results for the GSEA to calculate whether subtype-specific target genes of eRNAs are significantly affected by drug perturbations based on CMap. Different colored lines represent different breast cancer subtypes. The asterisk indicated P< 0.05.

Table S1. Demographic and clinical characteristics of TCGA cohort. Abbreviation: NA, not available.

Characteristics	Numbers of sample size(%)
Age	
<58	521 (0.475)
>=58	575 (0.524)
NA	1 (0.001)
SEX	
Female	1085 (0.989)
Male	12 (0.011)
ER	
Negative	238 (0.217)
Positive	808 (0.737)
Indeterminate	2 (0.002)
NA	49 (0.045)
HER2	
Negative	564 (0.514)
Positive	164 (0.149)
Indeterminate|Equivocal	191 (0.174)
NA	178 (0.162)
PR	
Negative	344 (0.314)
Positive	699 (0.637)
Indeterminate	4 (0.004)
NA	50 (0.046)
T classifcation	
T1|T2	916 (0.835)

T3|T4	178 (0.162)
TX	3 (0.003)
N classifcation	
N0|N1	880 (0.802)
N2|N3	197 (0.180)
NX	20 (0.018)
M classifcation	
M0	912 (0.831)
M1	22 (0.020)
MX	163 (0.149)
AJCC_PATHOLOGIC_TUMOR_STAGE	
Stage 1|Stage 2	804 (0.733)
Stage 3|Stage 4	269 (0.245)
Stage X	13 (0.012)
NA	11 (0.010)

Table S2. The result of univariate Cox regression analysis of clinical parameters and eRNAs in breast cancer.

Subgroup	samples	p.value	Hazard Ration(95% CI)
**SEX**			
Male	12		
Female	1082	0.8643	1.1872(0.1658-8.5039)
**medage**			
<58	520		
>=58	573	0.0003	1.8129(1.3087-2.5114)
**IHC_ER**			
Positive	806		
Negative	238	0.0978	1.3618(0.9448-1.9628)
**IHC_HER2**			
Positive	164		
Negative	562	0.0461	0.6086(0.3736-0.9915)
**IHC_PR**			
Positive	697		
Negative	344	0.093	1.3353(0.9529-1.8711)
**AJCC_M**			
M0	910		
M1	22	0	4.9087(2.9303-8.2226)
**AJCC_N**			
N0|N1	877		
N2|N3	197	0	2.3381(1.6003-3.4161)

**AJCC_T**			
T1|T2	913		
T3|T4	178	0.0036	1.7201(1.1944-2.4771)
**AJCC_STAGE**			
Stage 1|Stage 2	801		
Stage 3|Stage 4	269	0	2.6597(1.9007-3.7218)
**Enhancer**			
chr10:3848037-3849536	1094	0.0048	2.199 (1.272-3.8016)
chr10:5530637-5531333	1094	0.008	1.0475 (1.0122-1.084)
chr6:118759105-118759218	1094	0.0176	0.182 (0.0446-0.7426)
chr8:116990613-116990894	1094	0.032	1.9967 (1.0611-3.7572)
chr8:117175146-117175377	1094	0.0045	2.0527 (1.2504-3.3698)
chr8:117235182-117235423	1094	0.008	2.2821 (1.2399-4.2003)

Table S3. The information of genetic alteration-driven eRNAs associated with survival in breast cancer.

eRNAs	Differential Expression	Genetic alterations	Log-rank test using expression (P.value)	Log-rank test using copy number (P.value)
chr10:3848037-3849536	Upregulation	Copy number amplification	0.043	0.019
chr8:116990613-116990894	Upregulation	Copy number amplification	0.018	0.020
chr6:118759105-118759218	Downregulation	Copy number deletion	0.029	0.149
